# A qualitative study of senior hospital managers’ views on current and innovative strategies to improve hand hygiene

**DOI:** 10.1186/s12879-014-0611-3

**Published:** 2014-11-18

**Authors:** Elizabeth McInnes, Rosemary Phillips, Sandy Middleton, Dinah Gould

**Affiliations:** Nursing Research Institute, St Vincent’s Health Australia (Sydney) and Australian Catholic University, Executive Suite, Level 5, de Lacy Building, 390 Darlinghurst Road, Darlinghurst, 2010 NSW Australia; School of Nursing, Midwifery and Paramedicine, Australian Catholic University, 33 Berry Street, North Sydney, 2060 NSW Australia; School of Healthcare Sciences, Cardiff University, Eastgate House, Newport Road, Cardiff, CF24 OAB UK

**Keywords:** Hand hygiene, Qualitative interviews, Compliance, Hospital managers, Patient safety, Innovative strategies

## Abstract

**Background:**

Despite universal recognition of the importance of hand hygiene in reducing the incidence of healthcare associated infections, health care workers’ compliance with best practice has been sub-optimal. Senior hospital managers have responsibilities for implementing patient safety initiatives and are therefore ideally placed to provide suggestions for improving strategies to increase hand hygiene compliance. This is an under-researched area, accordingly the aim of this study was to identify senior hospital managers’ views on current and innovative strategies to improve hand hygiene compliance.

**Methods:**

Qualitative design comprising face-to-face interviews with thirteen purposively sampled senior managers at a major teaching and referral hospital in Sydney, Australia. Data were analysed thematically.

**Results:**

Seven themes emerged: *culture change starts with leaders, refresh and renew the message, connect the five moments to the whole patient journey, actionable audit results, empower patients, reconceptualising non-compliance* and *start using the hammer*.

**Conclusions:**

To strengthen hand hygiene programmes, strategies based on the five moments of hand hygiene should be tailored to specific roles and settings and take into account the whole patient journey including patient interactions with clinical and non-clinical staff. Senior clinical and non-clinical leaders should visibly champion and mandate best practice initiatives and articulate that hand hygiene non-compliance is culturally and professionally unacceptable to the organization. Strategies that included a disciplinary component and which conceptualise hand hygiene non-compliance as a patient safety error may be worth evaluating in terms of staff acceptability and effectiveness.

## Background

Hand hygiene is widely recognised as one of the most successful and cost-effective measures to reduce the incidence of healthcare associated infections (HCAIs) [1]-[3]. Hand hygiene best practice in clinical settings as a key preventive intervention has been endorsed by a number of key organisations including the World Health Organisation, Centers for Disease Control and Prevention, the National Health and Medical Research Council, Australian Commission on Safety and Quality in Health Care and Hand Hygiene Australia.

A systematic review conducted by the WHO on endemic HCAIs (1995-2010) found that of every 100 hospitalised patients at any given time, seven patients in developed countries and ten patients in developing countries will acquire a HCAI [4]. In Australia, there are approximately 200 000 HCAIs per year in acute health care facilities, making HCAIs the most common complication affecting hospitalised patients [5]. In Europe, it is estimated that HCAIs cause 16 million extra days of hospital stays, 37 000 attributable deaths and contribute to an additional 177 000 every year. In the US, approximately 99 000 deaths were attributed to HCAIs in 2002 and the annual economic impact in 2004 was estimated at approximately US $6.5 billion [4].

HCAIs can cause patients unnecessary pain and suffering, long term disability, excess deaths, increased resistance of microorganisms to antimicrobials [4] and as potentially preventable adverse events, prolong hospital admissions and place an avoidable and costly financial burden on the healthcare system [6]. Accordingly, the prevention and control of HCAIs has been identified as a national priority and is one of the National Safety and Quality Health Service (NSQHS) Standards (Standard 3) nominated by the Australian Commission on Safety and Quality in Health Care [7].

A plethora of strategies and interventions have been designed to improve compliance to best practice hand hygiene. Interventions include both single and multimodal interventions and fall into six main categories - resources (for example, installation of additional sinks, alcohol based hand wash), staff education and training, prompts or reminders, monitoring/audit and feedback, cultural and/or organisational change and campaigns [8]. Patient empowerment and involvement/engagement and sanctions for non-compliance are two newer approaches and emerging areas of hand hygiene research [9],[10]. However, despite the widely recognised importance of hand hygiene, considerable investment by governments, organisations and health facilities, and that hand-hygiene is a relatively simple act particularly with the availability of agents such as alcohol hand-rub, health care workers’ compliance with best practice has historically been variable (<40% to approximately 80% on average internationally) [10]-[12]. While research has been conducted in recent years to identify the most effective strategies for improving hand hygiene compliance [13],[14], a systematic review concluded that there is a dearth of high quality, robust evidence demonstrating which interventions are the most effective for improving and sustaining hand hygiene compliance [13]. Furthermore, “there remains an urgent need to undertake methodologically robust research” [13].

As a result of persistent challenges in achieving and sustaining hand hygiene compliance, clinicians, researchers and health care managers continue to search for new ways to address this important aspect of patient safety [15]. The health care system is complex and multidisciplinary, comprising senior executive personnel, clinicians, technical, support/service staff and administrators. Senior hospital managers are those clinical and non- clinical staff with delineated formal responsibility for administrating health care services and divisions, are involved in executive decision-making regarding budgets and staffing, and have responsibility for dealing with the outcomes of adverse events and for implementing systems level change [16]-[18]. As senior health care managers play an important role in patient safety and having input into the design and operationalization of quality improvement programs they can offer a unique perspective on how to improve areas of practice with which healthcare organizations are often struggling to improve [19].

Given their pivotal role, it is surprising that so little research has been conducted into the views of senior hospital managers in relation to patient safety strategies generally and hand hygiene more specifically [20],[21]. In addition, there is currently a debate in the infection control literature about whether there should be a shift in focus from systems failure to personal accountability because of criticisms that a systems approach has not resulted in optimal compliance [22]-[28]. To our knowledge, there have been no previous studies that have examined senior hospital managers’ perspectives on innovative strategies to improve hand hygiene compliance. Accordingly, our aims were to seek senior hospital managers’ views on current and innovative strategies to improve hand hygiene, whether failure to adhere to hand hygiene best practice should be conceptualised as a health care error and whether hand hygiene strategies would benefit from penalty-based strategies that focus on individual responsibility.

## Methods

### Study design

Qualitative face-to-face interviews.

### Setting

A 350 bed tertiary referral teaching hospital located in inner Sydney, Australia.

### Participants and recruitment

Staff were eligible to participate in the study if they held a position as a hospital executive, manager or clinical leader and had been employed for at least 12 months. Purposive sampling with snowballing was used to select participants to ensure that a range of views was captured from both clinical and non-clinical participants. Participants were selected on the basis of their seniority and ability to give an informed perspective on patient safety initiatives such as hand hygiene. An advanced letter outlining the research aims was sent informing potential participants of their selection and advising that the research team would make contact in order to organise an interview time should they be agreeable. A phone call was then made one week after initial contact and appointments made for the interviews. Participants who agreed to take part in the study were sent a copy of the participant information sheet and signed a consent form.

### Data collection

Individual semi-structured audio-taped face-to-face interviews of up to 30 minutes duration were conducted by one of the researchers. The interview guide was semi-structured and consisted of four open-ended questions with prompts and was piloted with minor revisions made. Participants were invited to give their suggestions of new strategies to improve hand hygiene compliance or ideas on strengthening existing strategies that are commonly employed in hospitals such as audit and feedback and education based on the 5 moments in hand hygiene. In addition, they were also asked for their views on whether hand hygiene non- compliance could be regarded as a type of health care error and their views on whether there is value in shifting from systems failure to personal accountability to improve compliance.

### Data analysis

Interviews were de-identified and audio recordings transcribed verbatim to produce transcripts of narrative text for thematic analysis. The coding frame was developed iteratively as two researchers read the transcripts and developed codes. Recurrent themes were noted including those covered by the topic guide and others that were raised by participants. Excerpts from the transcripts were allocated to these codes. After double coding each transcript was discussed by the two team members and the coding frame and themes were further refined by examining regularities, convergences and divergences in the data. The final set of themes emerged from this process and reflected the refined codes. Participant quotes were used to illustrate meaning in the themes and summaries. The final sample size was determined by saturation of themes, that is, no new insights were identified in the data.

### Ethics

Ethical approval was given by the St Vincent’s Hospital Sydney Human Research Ethics Committee [HREC Reference: HREC/1 f/SVH/76].

## Results

A total of 13 participants were interviewed. All who were approached accepted the invitation to participate in the study. Participants were senior clinical or non-clinical staff who held leadership positions that were administrative or clinical or a combination of both. The range of time that participants had worked at the hospital was 2 - 33 years. Those with clinical responsibilities came from medicine, nursing and allied health.

Thematic analysis of the transcripts of interviews revealed seven themes that reflect the strategies that participants thought are needed, either as novel strategies or modifications to existing programs:

Culture change starts with the leaders

Refresh and renew the message

Connect the five moments to the whole patient journey

Actionable audit result

Empower patients

Reconceptualising non-compliance

Start using the hammer

### Culture change starts with the leaders

A strategy thought important by many participants was a top-down approach towards embedding best practice at all levels of the organisation. To make hand hygiene part of the organisational mantra requires senior clinical and non-clinical leaders to visibly champion and mandate best practice initiatives as well as to articulate that non-compliance is culturally and professionally unacceptable:

*The culture is influenced by leadership of the organisation. It is everybody’s business If you don’t have the culture, you can have the best education program in the world, but it won’t be taken up at all if it isn’t supported by the leaders. (Senior Manager, Non-Clinical Support Services)*

Participants also felt that senior leaders should directly engage with frontline level managers (nurse unit managers for example) and secure their commitment to embed the message that best practice hand hygiene is *part of the organisational mantra and the way we do things around here. (Senior Manager, Clinical Services)* This could include mandating senior role models and champions to influence colleagues’ hand hygiene behaviours and drive cultural change at the ward/unit level through modelling hand hygiene behaviours and direct engagement with developing policies and procedures: *We have to somehow permeate that responsibility into the culture where people will say stop, I need you to wash your hands.* (*Senior Manager, Clinical Services)* Some participants suggested that an integral component of the clinical champions/role models’ function would be to identify personnel who repeatedly violate hand hygiene best practice:

*I know doctors have a lot of things on their minds, but this is a crucial, very simple thing you can do and I think we have got to start naming people. (Senior Manager, Clinical Services)*

Some participants extended this idea by stating that all clinicians should be empowered and mandated to remind or provide feedback to hospital staff observed not washing their hands, regardless of seniority and discipline:

*So, is the nurse watching the junior doctor? Is the patient watching the junior doctor? Is the cleaner watching the junior doctor? So everybody is a role model. (Senior Manager, Clinical Services)*

*I think the more people observe others doing it, the more they do it (Senior Manager, Clinical Services)*

While some participants emphasised that the hierarchical nature of the workplace and fear of repercussions such as hindering career advancement, would prevent widespread adoption of this strategy:

*They are very, very aware of the pecking order and they don't wish to provoke doctors, nurses, even though they feel like they see poor practices every day. (Senior Manager, Non-Clinical Support Services),*

others thought that changing culture necessarily entailed the risk of inter-disciplinary friction or conflict:

*That might create some conflict - do you know who I am? Yes, I do know who you are; you’re a member of the team. There might be some aggravation occasionally, but that’s how you change culture. (Senior Manager, Clinical Services)*

Training in ‘graded assertiveness’ was nominated to overcome this barrier:

*Give people the tools to maybe have that conversation. Words and phrases that people could use. (Senior Manager, Clinical Services)*

Overall, participants felt that a strategy that involved visible top-down championing of hand hygiene combined with the use of role models would strengthen existing hand hygiene initiatives by emphasising the organisational importance of best practice.

### Refresh and renew the message

Most participants expressed their strong belief that the standard approach of education, signage and reminders based on the WHO 5 moments, had reached a ceiling effect and had lost its impact and that key messages were now ‘stale’. Specifically, the content, focus and modes of delivery need to be reviewed and refreshed:

*We've got the good old five moments of hand hygiene posters that have been in the workplace now for 12/18 months.(Senior Manager, Non-Clinical Services)*

Some participants cited lessons from the advertising industry about the need for regular refreshing of the mode and content of messages:

*Posters that support hand hygiene best practice need to be revamped and changed in the same way that advertising posters get changed at my local bus stop. (Senior Manager, Non-Clinical Services)*

Some participants also questioned the evidence base underpinning hand hygiene educational and marketing strategies and the ability of these strategies to influence and change practice in the long-term:

*How do these strategies fit within the realm of known educational strategies and the literature about behaviour change? Are our signs on the wall, are our reminders the best way to educate people? Or is there a better way that could impact things? (Senior Manager, Clinical Services)*

An example given to support the view that educational strategies are insufficient for changing behaviour centred on the perceived infection risk and resultant hand hygiene behaviour of some clinical staff. Namely, that if a patient is perceived as low infection risk then clinical staff were observed to be not as compliant with hand hygiene best practice:

*If patients are considered a low risk, then I don’t think people are as compliant no matter what we do. (Senior Manager, Clinical Services)*

Another example given of the ineffectiveness of hand hygiene education was staff perceptions about the need to wear gloves in the belief that this would afford them extra protection from pathogen transmission from the patient:

*I think there is a bit of a gap in terms of knowledge of how hand-washing works and how it gets into crevices and that gloves are not so effective. They’re protecting themselves, quite frankly, before the patient. (Senior Manager, Non-Clinical Services)*

To reinvigorate educational programmes, some participants suggested the use of actual patient case studies, with ward rounds mentioned as an ideal opportunity for case-based education:

*It has to be real and it has to be real to the organisation you are working in. I do believe real case studies are what people remember. (Senior Manager, Clinical Services)*

Finally, linking hand hygiene assessment and education to professional college training and accreditation was suggested as a strategy to raise the profile of and reinforce the importance of hand hygiene.

### Connect the moments to the whole patient journey

The difficulty of applying the WHO five moments of hand hygiene in some clinical and non- clinical contexts and to the specific work or task being performed was noted by several participants:*...the hygiene interaction with a patient is not just clinical. (Senior Manager, Non-Clinical Services).* It was felt that an approach to improving compliance should be developed that takes into account the applicability of the 5 moments of hand hygiene to all settings and staff-patient encounters.

Non-clinical managers, whose staff are required to enter a ward but not to touch a patient, remarked that the five moments of hand hygiene could not always be followed:

*Frankly, when we saw the five moments posters... ... it didn’t mean much to us. We don’t touch patients but we enter the zone so two of those five were relevant. It’s just clouded the message for us. (Senior Manager, Non-Clinical Services)*

Similarly, clinical managers from allied health whose staff worked in non-acute patient settings (for example, outpatient rehabilitation groups rather than at the bedside) reported difficulties in adhering to the five moments of hand hygiene:

*You might have 10 patients in a group - it’s impractical to wash your hands in between each patient. (Senior Manager, Clinical Services)*

*You can’t possibly have five moments between each quick contact. So when we’re audited in those areas we have very low score rates (Senior Manager, Clinical Services)*

For these situations it was noted that there is no clear policy or guideline for hand hygiene practice.

*To be frank there doesn’t seem to be a very clear policy... . (Senior Manager, Clinical Services)*

To overcome some of these issues, it was suggested that hand hygiene principles, programs and education need to be developed that address the patient journey though-out hospital admission. Hand hygiene programs and policies should also be tailored to specific settings and different contacts with different clinical and non-clinical staff:

*For hand hygiene to be successful it must relate to the role and the work being done. It cannot be generic; it cannot ignore that the journey of a patient is not just clinical. (Senior Manager, Non-Clinical Services)*

It was suggested by some participants that future strategies could involve infection control staff. The latter could observe how different groups of staff interact with patients in various settings across the organisation to help identify how to introduce or modify the 5 moments in hand hygiene to accommodate the different settings and interactions:

*We need a solution as to how we might come up with hand hygiene model that keeps the patients safe and that suits these situations. (Senior Manager, Clinical Services)*

*Training sessions were conducted that connected the five moments to the non-clinical work we do. (Senior Manager, Non-Clinical Services)*

### Actionable audit results

All participants noted the reliance of hospitals on hand hygiene audits to measure hand hygiene performance. However, dissatisfaction was expressed with the method of reporting these results thus hindering the optimal use of such data as a lever for practice change. Participants stated that these reports were often over-simplified, a few months old and aggregated which meant that managers could not target specific hand hygiene issues and practices:

*What are we failing on here so that I’m really learning from it? We’re just getting the score and we just know we failed but what does that mean? Which particular action; what did the staff do or not do? (Senior Manager, Non-Clinical Services)*

Participants desired these data to be disseminated in a timely manner and to be specific to each department and clinical unit. Furthermore, data should be reported by discipline or job title, rather than by broad professional groupings. For example, physiotherapists and occupational therapists should be listed separately rather than grouped under ‘allied health’: *For those who are failing on hand hygiene they’re not getting any individualised feedback. (Senior Manager, Clinical Services)*. The aggregation of data was also perceived as contributing to a ‘blame culture’ between disciplines: *So we’re both pointing our fingers at one another; it’s always someone else’s problem. (Senior Manager, Non-Clinical Services)*.

Other important modifications that were mentioned that were needed included linkage of hand hygiene audit results to HCAI rates by ward in order to provide data on the beneficial effects of hand hygiene compliance and for there to be an effective mechanism to ensure that the results are filtered down to all staff:

*The only issue with that is whether it stops at the nurse unit manager and doesn’t go any further so the staff don’t actually see the results. (Senior Manager, Clinical Services)*

### Empower patients

A number of participants acknowledged that initiatives aimed at engaging and empowering patients to become more actively involved in their own care, could be extended to empowering patients to remind health care workers to wash their hands:

*If enough patients did that then that kind of embarrasses staff into it and eventually they just wouldn’t do it. I think it is probably the most powerful tool. (Senior Manager, Clinical Services)*

A hospital-wide approach in which staff were consulted about the best way to implement such a strategy, including staff education and giving patients the option of reminding staff to wash hands was considered feasible:

*If we give the patients the right to say, to a cleaner, a doctor, a visitor, anybody and say: I’m sick, you wash your hands, don’t give me anything else - and don’t get offended and cross with them for reminding - I believe that’s where we can move forward. (Senior Manager, Clinical Services)*

Staff would be required to undertake training in how to respond to patient requests:

*Give staff techniques - if a patient says to them, can you wash your hands, that they don’t get defensive; that they say, I have washed my hands, but I’ll go out and wash them again for you, to make you feel more comfortable. (Senior Manager, Clinical Services)*

Several participants reflected that clinicians may react negatively to patient reminders and that patients may be reluctant to remind staff for fear of the effect this may have on their relationship with health care staff and the care they receive:

*Some clinicians might respond, okay well, sorry, I’m actually very busy, that’s right I forgot. But some might object and not give good care regardless. (Senior Manager, Clinical Services)*

Although some participants spoke in terms of patient rights to prompt clinical and non- clinical staff to wash their hands, others were more circumspect. This was largely because they felt that putting the onus on the patient would place patients who were already vulnerable in a difficult position.

### Reconceptualising non-compliance

While participants were in agreement about the importance of hand hygiene as a patient safety issue: *if you’re not washing your hands, you’re flouting a very basic patient safety tenet (Senior Manager, Clinical Services)*, there were differences in views about whether hand hygiene non-compliance should be defined as a health care error.

*I don’t think they [clinicians] see that as an error. They’d probably see it as non- compliance, but not as an error per se. (Senior Manager, Non-Clinical Services)*

Other participants stated that hand hygiene non-compliance should be construed as a health care error:

*If there is a widely accepted view that this (hand hygiene) needs to be done and it’s not been done, then not doing it could fall into the error category. (Senior Manager, Clinical Services)*

Several participants viewed non-compliance to hand hygiene in the same light as a staff member ‘causing an injury’ to a patient and that non-compliance placed patients in danger as it could potentially cause a critical illness as well as breached local and NSW Ministry of Health policy. Parallels were drawn between an immunocompromised patient getting an infection through poor hand hygiene practice and a patient being given the wrong medication and experiencing an adverse event. Some participants thought that staff who repeatedly do not wash hands should be considered as being unsafe to practice, be held personally accountable for their actions and reported to the appropriate registration boards.

However, other participants were not comfortable with defining hand hygiene non- compliance as a health care error and rationalised non-compliance as resulting from lack of time and lack of awareness:

*How could I discipline a staff member’ They take shortcuts because they’re running behind time or something. (Senior Manager, Non-Clinical Services)*

*I don’t think it’s conscious, refusing to (wash hands). (Senior Manager, Clinical Services)*

The question was raised by participants as to how breaches would be documented: *The problem is that it could be so common, but nobody is going to fill out an incident form for breaching hand hygiene. (Senior Manager, Clinical Services)*. It was also stated that current hospital incident reporting mechanisms have not been designed for recording patient safety breaches such as poor hand hygiene practice and that a specially designed reporting system would be needed. In addition, it was felt unlikely that staff would complete incident forms for hand hygiene lapses because the latter were thought to be common.

### Overdue to start using the hammer

Opinions differed over whether the introduction of penalties (such as financial penalties, linking professional accreditation and re-accreditation to successful completion of a hand hygiene education program; and performance management) for repeated non-compliance, would be an appropriate strategy, particularly in light of the prevailing no blame culture in health. Some participants indicated that they did not favour ‘overly heavy handed disciplinary action’ because of the negative connotations of such an approach and its potentially negative impact on building a ‘positive’ culture:

*It’s more about let’s do the right thing, rather than punish you for the wrong thing....-*

*a cultural approach of positivity. (Senior Manager, Non-Clinical Services)*

Some also commented that a penalty-based approach could affect staffing levels and queried how appropriate penalties would be determined:

*There aren’t enough nurses and there aren’t enough doctors’.It’s very hard to think of a punishment for a health professional. (Senior Manager, Clinical Services)*

However, some participants supported the introduction of penalties, saying that it was overdue in the light of persistently low compliance rates and the risk of HCAIs: *I agree that penalties should be introduced. We’re all health professionals, We’ve all supposedly had training. Everyone knows how important hand hygiene is. (Senior Manager, Clinical Services)*.

Taking disciplinary action for hand hygiene violations was *the elephant in the room that’s never been addressed. (Senior Manager, Non-Clinical Services).* Participants who favoured a penalty-based strategy however, believed it should be developed by the NSW Ministry of Health in consultation with hospitals. The strategy should clearly define what constitutes non- compliance and articulate the penalties for repeated non-compliance:

*I have no hesitation in penalising people for that. If they’ve been through a process that’s fair and been given a warning and further education. (Senior Manager, Clinical Services)*

There were suggestions that a managerial approach such as performance management could assist with a shift from system responsibility to individual accountability and that performance management was an under-utilized approach:

*I cannot get my head around the fact that there seems to be very little performance management in relation to hand hygiene - even to the extent of disciplinary action being taken. We’re talking about professional practices. Considering how high the stakes are I just can’t understand how it’s taking so long. I think we are overdue to actually start to use a hammer. (Senior Manager, Non-Clinical Services)*

Mandates and support from hospital executive for implementing performance management would be necessary to make such an approach effective and consistent:

*Performance management is something that managers typically shy away from. Only when their hand is really pushed will they follow up on it. (Senior Manager, Non- Clinical Services)*

All participants agreed that a graded/staged or tiered approach to managing non-compliant staff, that is, penalties or disciplinary measures increasing in severity with each occasion of breach of hand hygiene policy, would be the preferred approach. Thus, the first level of the disciplinary process could involve individuals being given a warning and remedial education; repeated failure could lead to a disciplinary measure where the individual would meet with the infection control nurse or their direct line manager and be asked to explain their non- compliance and the final level could involve suspension from clinical practice until the individual could demonstrate compliance with hand hygiene, or at its most severe, dismissal. A number of participants also argued that repeated non-compliance with hand hygiene should be a notifiable offence, with a mandatory requirement to report to the relevant registration board:

*The remit of the registration boards is public safety and this is a public safety issue. I think very few people would get to that point, but unless you escalate it or elevate it to that level, people will continue to unconsciously flout it. (Senior Manager, Clinical Services)*

Many voiced the view that a penalty-based strategy would be more acceptable if it was part of a prolonged, multipronged approach. As articulated by one participant:

*It’s like everything, a multi-pronged attack, it’s not just one thing that’s going to do it you’ve got to do a number of different things and you’ve got to keep the pressure on and name people and have some disincentive not to do it, not to comply. (Senior Manager, Clinical Services)*

## Discussion

To our knowledge, this is the first study to examine the views of senior hospital managers’ perspectives and suggestions for strategies to address hand hygiene non-compliance in the acute care setting. This study was conducted at one large metropolitan acute care hospital and the views expressed by these participants may not necessarily be representative of all senior managers either at this hospital or other hospitals. The study provides insights rather than findings that can be generalised. Senior clinical and non-clinical managers were interviewed because they can provide a unique ‘helicopter’ view across organisational levels and also because of their involvement in patient safety initiatives. Future studies could examine the views of other groups of hospital staff and investigate the acceptability by different disciplines of some of the suggested strategies. Participants were asked to comment generally on hand hygiene campaigns and programs; this qualitative study was not linked to an evaluation of the impact of a particular hand hygiene program. Managers from a diverse range of professions and disciplines were included and the semi-structured interview guide allowed for the expression of a broad range of views. Given this diversity, it is interesting to note that there was considerable consistency across clinician and non-clinician participants’ responses in relation to some of the themes. For example, the need for senior managers of all levels to lead the way in terms of culture change; for messages and education to be reinvigorated; for audit results to be timely and de-aggregated and also for hand hygiene strategies based on the 5 moments of hand hygiene to be tailored to particular settings. While there were concerns expressed by some participants from the clinical manager group about empowering hospital staff to challenge poor hand hygiene practice, there was acknowledgement that training and hospital-wide support could overcome some of the perceived barriers to this approach. Views were ambivalent about whether hand hygiene lapses should be categorised as patient safety errors. However, there was some receptiveness to the idea of at least debating how repeated lapses should be penalised and there was enthusiasm across the sample for introducing the notion of individual responsibility for ensuring best practice hand hygiene.

There were a number of important findings from this study that may assist with the development of future hand hygiene programmes in hospitals. Firstly, that the drive for change has to come from the `top-down’ but not just in the form of policies and protocols. The theme *Culture change starts with the leaders* reflected the need for both top-down and bottom-up sharing of responsibility to make hand hygiene best practice an organisational priority and for embedding it into the practice culture. The visible and sustained commitment by managers to lead by example and engage directly with middle managers such as nursing unit managers to embed change and implement the mantra `that is what we do here’ is required. This view is reflected in the description that an organisation’s culture is not something that it has, but is something “that an organisation is” [29]. Furthermore, a culture of safety is created when there is shared commitment for promoting and encouraging safety promoting behaviours [30]. The value, priority and commitment that is attached to a patient safety issue by senior staff is important in galvanising an organization toward patient safety goals [19],[31]. Strategies such as leading by example - ‘the executive walk-around’ which includes modelling of hand hygiene best practice by executive managers have been reported as an effective strategy engaging frontline staff [30],[32] which can foster ‘collective mindfulness’ across all organisational levels of the importance of best practice [33].

Some participants from the clinical manager group were enthusiastic about trialing in the future the use of role models to champion hand hygiene and influence clinician behaviour particularly if there is hospital-wide training and support in place. However, this strategy has been evaluated with mixed results [34]-[36]. For example, in one study nurses’ hand washing intentions were influenced by peer pressure from physicians and administrators [37] while another study reported that peer feedback on hand hygiene performance whilst effective in the intervention period, was not sustainable [38]. In terms of willingness of clinicians to remind those from other disciplines or who are more senior to wash their hands and impact of this approach there has been some research. In a survey of doctors, less than half the sample responded that they would be willing to prompt another doctor to perform hand hygiene and this willingness decreased as the seniority of the observed doctor increased [39]. However, a study that evaluated the impact of medical students as hand washing champions to remind physicians to wash their hands, reported that physician compliance with hand hygiene improved from 68% to 95% within 6 months and that physicians readily accepted reminders [36]. Similarly, another study that included encouraging senior health care professionals to serve as role models and encourage junior health care professionals to comply with hand hygiene protocols reported improvements in hand hygiene compliance and a decrease in the incidence of nosocomial infections, however it was not possible to identify which component of the educational program (5 elements) was most effective [34].

Senior managers, particularly non-clinical and clinical staff who work in group settings, believed that as well as needing to refresh hand hygiene messages and educational strategies, a ‘one size fits all’ approach to hand hygiene is unworkable in some non acute/outpatient care settings such as in physiotherapy groups and for patient encounters with non-clinical staff, such as food and environmental services staff. This finding is in accordance with the current literature which shows that for complex health care settings, that are comprised of numerous units and teams, each with its own micro-culture and work practices, organization-wide hand hygiene programs may not successfully transfer or suit each of these environments [40],[41].

Hand hygiene education has been largely developed with bedside clinicians as the target audience. This can have unintended consequences such as non-clinical staff having difficult applying hand hygiene principles or discounting important hand hygiene messages as irrelevant to them. There is therefore a need to develop strategies that take into account the entire patient journey and which can be adapted to and tailored for differing workplace roles, responsibilities, disciplines and settings, level of patient contact, and level of knowledge about infection control [8],[42],[43]. The need to adapt the 5 moments to specific situations and needs has been previously flagged in earlier studies [44], illness severity [45]. A study reported that hand hygiene compliance increased from an average of 70% to 97% following an intervention that identified practical ways of incorporating the 5 moments in hand hygiene for health care workers [42]. Thus, involving hospital staff in initiatives to map the five moments in hand hygiene to the different clinical and non-clinicians interactions and settings that patients experience during hospital admission may enable the development of tailored solutions.

Empowering patients to remind staff to wash their hands has been suggested as part of an overall strategy to facilitate active patient involvement in their health care management [46]-[49], although patient empowerment has been more commonly used in relation to chronic disease management rather than in the acute care setting [8]. In Australia, the Clinical Excellence Commission has actively promoted patients and visitors’ involvement in hand hygiene through campaigns such as *“It’s OK to ask”* or remind hospital staff to wash their hands [50]. However, while some clinical and non-clinical managers were supportive of trialling patient empowerment to remind staff to wash hands, they also identified a number of potential barriers to implementing this strategy that have been reflected in research in this area. These included patient-related factors such as cultural and socioeconomic background, frailty, vulnerability and illness severity [9],[51],[52] and, the need to have a programme of health care worker preparedness [46]. Research has found that some patients may be reluctant to speak up for fear of reprisal through sub-optimal care or of being seen as a ‘difficult’ patient [51]. A survey of acute care patients found that patients believe they should be able to trust that they are being provided with safe, competent care whilst in hospital, rather than take on an active role in ensuring their safety [53]. Studies have also found mixed views regarding the acceptability by health care workers about patient involvement in hand hygiene reminders [46],[54]-[58], that health care workers do not believe that patients should assume this role [9] and that tensions between patients and health care workers may arise [46]. An alternative way of introducing a patient focus into hand hygiene campaigns that was recommended by senior clinical managers, was using local patient case studies in education or ward rounds to vividly illustrate the consequences for patients of sub-optimal hand hygiene practice.

Despite evidence that audit and feedback interventions in health care are effective in improving compliance with a desired behaviour [59], both clinical and non-clinical participants in our study were dissatisfied with several components of hand hygiene audit and feedback strategies. Specifically they reported that the aggregation and presentation of the data did not allow them to make practical and meaningful use of it. De-aggregation of data was strongly recommended to enable managers to readily identify professional groups and individual staff members who need to be targeted for further hand hygiene education. The time-lag between the collection of hand hygiene audit data and the dissemination of results to the unit/department manager level and then to front-line clinicians was also an issue. These findings are consistent with research that has shown that compared to low-performing health facilities, high performing facilities deliver feedback in a timely and individualised manner; conversely low performing facilities were variable in their timeliness relying on standardised facility level reports as a source of feedback [60]. Furthermore, a recent critical review of the Australian National Hand Hygiene Initiative has highlighted the financial burden placed on hospitals participating in national audits and the low return in terms of improvement in hand hygiene compliance [43]. The authors argue that it is “time to move from our obsession with auditing” and refocus hand hygiene programs to deliver targeted interventions based on local gaps in practice [43].

Clinical senior managers were unanimous in their agreement that it is time to debate if hand hygiene non-compliance constitutes a patient safety error. However, there was difficulty for some participants in characterising non-compliance as a health care error. This reflects the complexity in broadening conceptualisations of safety errors to include a range of health care practices not just those traditionally conceptualised as such, for example surgical errors and medication errors [61]. Some participants from the clinical senior manager group were uncomfortable with attributing non-compliance to a conscious decision or deliberate intent and also pointed to the difficulty in showing that an individual’s non-compliance with hand hygiene directly causes harm to a patient. However overall, there was agreement that it is time for the balance of responsibility to shift from a sole focus on organisational/system failure to a focus that includes personal accountability where systems and resources are in place to support best practice. For this shift to occur, participants recommended that clear policies and guidelines are needed with input from key stakeholders, that are backed with organisational support at all levels, and that includes an appropriate reporting mechanism and criteria for determining when hand hygiene lapses would be recorded as a patient safety error.

The reluctance among some participants from the clinical senior manager group, to support a penalty-based approach could be linked to the current ‘no blame’ culture of incident reporting. It has been recently noted that the ‘no blame’ culture has resulted in the concept of individual responsibility being largely removed from discussions of patient safety [27]. However, others, believed that it was time to evaluate a penalty-based approach which could consist of performance management, compulsory education, and as a last resort suspension from practice. However, they were not generally supportive of financial penalties and equivocal about other penalties such as loss of credentials/privileges and dismissal for repeated failure to wash hands, strategies that have been trialled in the United States with varying degrees of success [62]-[67]. Lastly, all participants stated their belief that, regardless of whether a penalty-based approach is adopted, a multi-modal approach produces better outcomes than a single intervention. Those clinical senior managers who did support a penalty-based approach recommended that it be situated within local quality and safety frameworks for incident reporting and management an approach that has been recommended by others [24]. This is consistent with previous research that has demonstrated that an integrated, bundled or multimodal approach to behaviour change is more efficacious than a single intervention [42],[46],[49],[66],[67].

There is now a strong body of literature establishing that senior managers in acute care health services have responsibility for, and play a pivotal role in, ensuring the success of quality and safety programs [16]-[19]. This study addressed a gap in the literature by exploring the views of senior managers on current and future strategies to improve hand hygiene compliance. It is of interest to note that a number of the participants in this study could be described as ‘hybrid managers’ [68] in that they had clinical backgrounds and held positions in the organisation at the interface between professional frontline and managerial domains. There is increasing awareness that these managers are uniquely placed within a health care organisation to broker the sources of knowledge and information necessary to improve patient safety. The innovative strategies that were nominated by participants provides a compelling case to conduct further research into ways of utilising managers’ knowledge based capabilities to improve patient safety.

## Conclusions

From the perspective of hospital clinical and non-clinical senior managers, there are several suggestions for strengthening and reinvigorating hospital hand hygiene strategies. First, visible and on the ground organisational support and leadership including role modelling and secondly tailoring the five moments of hand hygiene to specific roles and settings, including interactions with non-clinical staff, that take into account the entire patient hospital journey. Future research is needed on frontline hospital staff’s views of the hand hygiene strategies nominated in this study, including further investigation of whether there is value in conceptualising hand hygiene non-compliance as a patient safety error and with implementing penalty-based approaches.
